# Photodynamic therapy outcome modelling for patients with spinal metastases: a simulation-based study

**DOI:** 10.1038/s41598-021-97407-z

**Published:** 2021-09-09

**Authors:** Abdul-Amir Yassine, William C. Y. Lo, Tina Saeidi, Dallis Ferguson, Cari M. Whyne, Margarete K. Akens, Vaughn Betz, Lothar Lilge

**Affiliations:** 1 Edward S. Rogers Sr. Department of Electrical and Computer Engineering, University of Toronto, Toronto, ON M5S 3G8 Canada; 2 Harvard Medical School, Boston, MA 02115 USA; 3 Division of Health Sciences and Technology, Harvard-Massachusetts Institute of Technology, Cambridge, MA 02142 USA; 4 Department of Medical Biophysics, University of Toronto, Toronto, ON M5G 1L7 Canada; 5 Institute of Biomedical Engineering, University of Toronto, Toronto, ON M5S 3G9 Canada; 6 Orthopaedic Biomechanics Laboratory, Sunnybrook Research Institute, Toronto, ON M4N 3M5 Canada; 7 Department of Surgery, University of Toronto, Toronto, ON M5G 1L7 Canada; 8 Holland Bone and Joint Research Program, Sunnybrook Research Institute, Toronto, ON M4N 3M5 Canada; 9 Techna Institute, University Health Network, Toronto, ON M5T 1P5 Canada; 10 Princess Margaret Cancer Center, University Health Network, Toronto, ON M5G 1L7 Canada

**Keywords:** Computational biophysics, Preclinical research, Software, Biophotonics

## Abstract

Spinal metastases often occur in the advanced stages of breast, lung or prostate cancer, resulting in a significant impact on the patient’s quality of life. Current treatment modalities for spinal metastases include both systemic and localized treatments that aim to decrease pain, improve mobility and structural stability, and control tumour growth. With the development of non-toxic photosensitizer drugs, photodynamic therapy (PDT) has shown promise as a minimally invasive non-thermal alternative in oncology, including for spinal metastases. To apply PDT to spinal metastases, predictive algorithms that optimize tumour treatment and minimize the risk of spinal cord damage are needed to assess the feasibility of the treatment and encourage a broad acceptance of PDT in clinical trials. This work presents a framework for PDT modelling and planning, and simulates the feasibility of using a BPD-MA mediated PDT to treat bone metastases at two different wavelengths (690 nm and 565 nm). An open-source software for PDT planning, PDT-SPACE, is used to evaluate different configurations of light diffusers (cut-end and cylindrical) fibres with optimized power allocation in order to minimize the damage to spinal cord or maximize tumour destruction. The work is simulated on three CT images of metastatically involved vertebrae acquired from three patients with spinal metastases secondary to colorectal or lung cancer. Simulation results show that PDT at a 565 nm wavelength has the ability to treat 90% of the metastatic lesion with less than 17% damage to the spinal cord. However, the energy required, and hence treatment time, to achieve this outcome with the 565 nm is infeasible. The energy required and treatment time for the longer wavelength of 690 nm is feasible (${\sim }\,40$ min), but treatment aimed at 90% of the metastatic lesion would severely damage the proximal spinal cord. PDT-SPACE provides a simulation platform that can be used to optimize PDT delivery in the metastatic spine. While this work serves as a prospective methodology to analyze the feasibility of PDT for tumour ablation in the spine, preclinical studies in an animal model are ongoing to elucidate the spinal cord damage extent as a function of PDT dose, and the resulting short and long term functional impairments. These will be required before there can be any consideration of clinical trials.

## Introduction

Spinal metastases, which are particularly prevalent in advanced stage breast, prostate, and lung cancers, remain a major clinical challenge despite existing treatment options. This is often due to the genetic variability of tumours making them resistant to systemic and localized therapies^1–4^. Patients with spinal metastases often suffer from skeletal-related events including debilitating pain (sometimes requiring very high doses of potent analgesic drugs) and pathologic fractures, which may lead to neurological symptoms. Such symptoms include spinal cord or nerve root compression which may result in motor or sensory deficits and bowel or urinary incontinence in severe cases, significantly impacting quality of life. Patients with advanced metastatic disease are typically poor surgical candidates, and receive treatment aimed primarily at palliative pain control.

Current treatment options for spinal metastases include localized, targeted approaches such as surgical stabilization (for cases with vertebral instability requiring cord decompression)^2^ and radiation and thermal therapies^3,5^ as well as systemic treatments such as chemotherapy, immunotherapy and bisphosphonates^4^. While systemic treatments like bisphosphonates have shown benefit in relieving metastatic bone pain and delaying complications, they usually cause adverse effects on the gastrointestinal and haematopoietic system^6^. Conventional radiation therapy has been shown to achieve an overall response rate (palliation of pain symptoms) of approximately 60%, and complete abrogation of pain (complete response rate) in only ${\sim }\,$25% of patients^7^. In contrast, focal radiation therapy (i.e. stereotactic body radiation therapy, SBRT) is a well-established non-invasive approach that precisely targets metastatic lesions in bone and provides pain relief in the majority of patients ($>90\%$). Thibault et al.^8^ reported that patients undergoing SBRT had sustained pain relief of 86% and local control of 88% (using CT criteria) at a median follow-up of 21 months. Yet, SBRT’s repeated use is limited by toxicity to the spinal cord (radiation-induced myelopathy) and an incidence of fracture post treatment^9–12^. Additionally, SBRT requires sophisticated hardware and software for treatment planning, patient setup, and careful patient selection^13^, which currently limit its widespread use.

Radiofrequency ablation (RFA) is also utilized clinically for localized treatment of spinal metastases^5^. RFA is a thermal modality that utilizes high-frequency alternating current (by placing needle electrodes into the surrounding tissues) to heat and eventually ablate tumours. RFA may also be coupled with vertebral cement augmentation (VCA) to provide vertebral stabilization and extended pain relief. Mayer et al.^14^ recently reported that 80% of patients who underwent bipolar RFA with VCA achieved favourable pain relief (3 points reduction on the visual analogue scale, VAS) at a mean follow-up of 3.4 months. Neurologic injuries during RFA of spinal metastasis may occur if performed too close to critical structures, as such RFA generally is limited for tumours in the posterior vertebral body an in cases with a breach of the posterior vertebral body wall^5^.

Photodynamic therapy (PDT) is an emerging non-thermal minimally invasive modality that offers the potential to precisely target spinal metastasis. In preclinical rodent models, PDT has been shown to successfully ablate spinal metastases and improve vertebral mechanical stability with increased osteoid formation, particularly when combined with systemic bisphosphonates^15–19^. A recent phase I trial further demonstrated the safety and feasibility of BPD-MA mediated (Benzoporphyrin derivative mono-acid photosensitizer, trade name: Visudyne, Novartis, QC, Canada) PDT as a tumour-ablative adjunct modality prior to VCA in patients with spinal metastases^20^. The study evaluated various treatment parameters including the energy delivered and drug-light interval. The results suggested that vertebral PDT as an adjunct to VCA is safe from a pharmaceutical and neurological perspective. The 50 J cm$^{-1}$ and 100 J cm$^{-1}$ treatment groups showed a clinically significant reduction in pain^20^.

A potential advantage of PDT over radiation or thermal therapies is the ability to repeatedly treat the same site^21^, without risking toxicity to the spinal cord or other critical structures. High light scattering in intact vertebral bone confines the excitation photons and limits the light reaching the spinal cord. In cases of posterior vertebral body metastatic involvement, particularly if the cortical shell is compromised, establishing light scattering through the tumour and remaining bone is critical to safety. For this, personalized PDT treatment planning could be used to reduce any potential injury risk in targeting the malignancy and avoiding impact to adjacent spinal cord or nerve roots. Yet, PDT treatment planning is challenging due to the lack of established 3D modelling tools and framework for optimizing the source configuration. Such planning requires visualizing the final fluence distribution and evaluating the quality of the resulting treatment plan in a highly heterogeneous geometry.

Here, we investigate the feasibility of BPD-MA mediated PDT^22^ in patients with spinal metastases by presenting a framework for PDT treatment modelling and simulation leading towards systematic PDT planning. We incorporate the complexity and heterogeneity of the 3D tumour geometry using original pre-treatment CT datasets and contours for patients treated with SBRT. Using an interstitial PDT (iPDT) planning optimization tool called PDT-SPACE^23,24^, the optimal source power allocation for two types of light sources embedded within the metastatic lesion is determined, demonstrating the ability to tailor the light dose (3D fluence distribution) to the tumour geometry while minimizing damage to the spinal cord using PDT dose threshold values (considering a PDT threshold model^25^) derived from preclinical models. We simulate the attainable efficacy of PDT at two different activation wavelengths,  690 nm and 565 nm. Both of these wavelengths are peak absorbance bands for BPD-MA^26^. We report the predicted tumour coverage and damage to the spinal cord in both cases.

**Figure 1:**
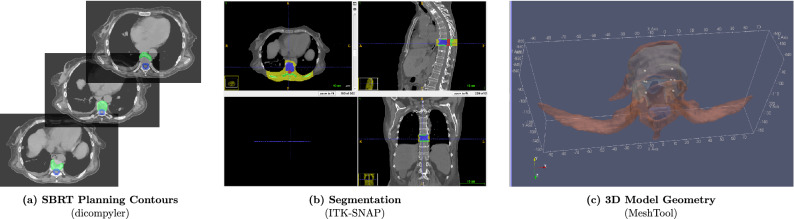
Overview of PDT treatment planning framework for a female patient with a metastatic T8 sclerotic lesion: (**a**) extraction of contours from stereotactic body radiation therapy plan using the Dicompyler tool, including the clinical target volume or metastasis (green) and spinal cord (blue), (**b**) segmentation of the original CT dataset using ITK-SNAP to delineate the metastasis (purple), normal bone (green), spinal cord (red), and muscle (yellow) at the T8 level, (**c**) generation of the 3D mesh geometry using MeshTool for light dose simulation with FullMonte Methods.

## Methods

### Treatment planning framework

Computer tomography (CT) images of patients with spinal metastases were used to generate the virtual PDT treatment plans used in this work (Fig. 1). Patient identifiers were removed prior to using these imaging datasets from three patients with spinal metastases treated with stereotactic body radiation therapy (Department of Radiation Oncology, Sunnybrook Health Sciences Centre, Toronto, ON, Canada). This is a retrospective study utilizing the imaging datasets. Informed consent from the patients was obtained. The study followed all applicable guidelines and regulations. The study was approved by the Sunnybrook Health Sciences Ethics Review Board (Toronto, ON, Canada). Table 1 describes the three cases along with their locations. Guided by the contours from the SBRT plan, target structures and organs at risks were segmented using ITK-SNAP^27^, including the metastasis, spinal cord, normal surrounding bone, and muscle. Note that the latter two structures were manually segmented as they were not explicitly contoured. Other surrounding structures were omitted as they are not expected to be significantly impacted by PDT, because they were distal to the target and were exposed only to a low photon density, or they have a limited vasculature and hence do not take up the photosensitizer. The segmentation data was used to generate surface meshes which were subsequently converted to the mesh geometry (using a custom-made tool called MeshTool (available at https://gitlab.com/FullMonte/Meshtool) required by the FullMonte light dosimetry software^28^ (available at https://gitlab.com/FullMonte/fullmontesw), and the PDT-SPACE optimization software (available at https://gitlab.com/FullMonte/pdt-space).

**Table 1:** The metastatically involved vertebrae utilized in this study.

Case	Lesion location	Description	Treatment date
1	T8	Metastatic colorectal cancer—sclerotic	Jan. 2019
2	C7	Metastatic non-small-cell lung cancer—lytic	Jan. 2019
3	T8	Metastatic EGFR positive non-small-cell lung cancer—lytic	Sept. 2018

PDT-SPACE^23,24^, an open-source optimization software for iPDT treatment planning, was used to optimize the power allocation across the source geometry, including a scenario of 4 cut-end fibres with a 400 $\upmu$m core diameter and $NA =0.22$ (placed into the spinal metastasis, pointing diagonally at $45^{\circ }$ away from the spinal cord) as well as a scenario of two cylindrical diffusers with a 500 $\upmu$m radius inserted diagonally into the spinal metastases. Different weights were applied (called *tumour weight*) to drive the optimization algorithm towards favouring tumour targeting at the expense of critical structures such as the spinal cord or favouring no damage to the spinal cord at the expense of less damage to the tumour. Dose-volume histograms were generated for each tissue type by PDT-SPACE to assess the given PDT plan’s quality. The final PDT treatment plan at two different activation wavelengths ($\lambda _1 = 690$ nm and $\lambda _2 = 565$ nm) with the corresponding iso-fluence contours are visualized using ParaView 5.6.0^29^.

**Table 2:** Tissue optical properties for each region in the 3D model at two activation wavelengths ($\lambda _1 = 690$ nm and $\lambda _2 = 565$ nm).

Tissue	$\lambda _1 = 690$ nm				$\lambda _2 = 565$ nm			
	$\mu _s$ (mm$^{-1}$)	$\mu _a$ (mm$^{-1}$)	*g*	*n*	$\mu _s$ (mm$^{-1}$)	$\mu _a$ (mm$^{-1}$)	*g*	*n*
Spinal cord	15.47	0.0216	0.9	1.41	22.48	0.108	0.9	1.41
Bone	15.23	0.01	0.9	1.56	32.09	0.04	0.9	1.56
Sclerotic metastasis$^{\mathrm{a}}$	15.23	0.01	0.9	1.56	32.09	0.04	0.9	1.56
Osteolytic metastasis	16	0.009	0.9	1.56	10.22	0.09	0.9	1.56
Muscle	7.356	0.052	0.93	1.41	11.61	0.36	0.93	1.41

$^{\mathrm{a}}$Assumed to have similar tissue optical properties as the bone.

**Table 3:** PDT dose threshold values for each region in the 3D model.

Tissue	*T* ($\times 10^{18}$ photons/cm$^3$)	PS uptake ($\upmu$g/g)	$\Phi _{threshold}$ (J mm$^{-2}$)
Spinal cord	0.1	0.13	0.02
Bone	1	0.3	0.1^a^
Metastasis	10	0.7	0.4
Muscle	1	0.13^b^	0.1

$^{\mathrm{a}}$Estimated based on a preclinical study in a porcine model^30^.

$^{\mathrm{b}}$Assumed to be similar to the spinal cord.

### Model specification: optical properties and dose constraints

Table 2 summarizes the tissue optical properties (absorption coefficient $$\mu _a$$, scattering coefficient $$\mu _s$$, anisotropy *g*, and refractive index *n*) at the two activation wavelengths. The optical properties of the osteolytic tumours, spinal cord, bone, and muscle are based on literature values^31–36^ (interpolated for 565 nm), while those of the sclerotic spinal metastasis are assumed to be similar to the bone. Table 3 summarizes the PDT dose constraints considered in the current model. The spinal cord is assigned 1/10 the PDT dose threshold of normal bone, while the metastasis is assumed to be 10× more resistant than normal bone. Based on the photosensitizer uptake data (BPD-MA at 15 min with a dose of 2 mg/kg) from an earlier preclinical study^37^, the threshold fluence,$$\Phi _{threshold}$$ (J cm$$^{-2})$$, at the boundary of necrosis is computed using the PDT threshold formula^38^ shown in Eq. (1).

1 $$\begin{aligned} \Phi _{threshold} = \frac{hc_0T}{2.3\varepsilon C\lambda } \end{aligned}$$

where $h=6.626\times 10^{-34}$ Js is Planck’s constant, $c_0\approx 3\times 10^9$ ms$^{-1}$ is the speed of light in vacuum, $\varepsilon$ is the photosensitizer’s molar extinction coefficient (($\upmu$g/g)$^{-1}$ cm$^{-1}$), *C* the concentration or uptake in the tissue ($\upmu$g/g), *T* is the necrosis threshold dose in the number of photons absorbed per cm$^3$ and $\lambda$ is the wavelength of light used to activate the photosensitizer. For BPD-MA, the molar extinction coefficients are 33000 M$^{-1}$ cm$^{-1}$ or 45030662 ($\upmu$g/g)$^{-1}$ cm$^{-1}$ and 6724 M$^{-1}$ cm$^{-1}$ or 10082842 ($\upmu$g/g)$^{-1}$ cm$^{-1}$ at 690 nm and 565 nm, respectively^39^. Here we provide $\Phi _{threshold}$ in (Jm m$^{-2}$) units to which clinicians are more familiar with.

### Ethics approval and consent to participate

This is a retrospective study utilizing imaging datasets obtained from patients for whom informed consent was obtained. The study followed all applicable guidelines and regulations. The study was approved by the Sunnybrook Health Sciences Ethics Review Board (Toronto, ON, Canada).

## Results

Using the final 3D mesh geometries and the dose constraints specified in Table 3, two light source configurations were evaluated to demonstrate the use of the 3D PDT treatment planning framework for the first T8 metastatic lesion in Table 1. For this model, the volumes of the different tissue types in the 3D model are as follows: spinal metastasis is 23.21 cm$^3$, the spinal cord is 4.72 cm$^3$, normal bone is 31.14 cm$^3$ and the muscle is 171.84 cm$^3$. The corresponding iso-fluence contours based on the optimized power allocation are shown to compare the quality of the predicted treatment outcomes for all scenarios. Additionally, we present dose-volume histograms (DVHs) to see effect of both wavelengths on the spinal cord damage. In the first set of optimization simulations the power allocation was optimized to attain at least 90% necrosis in the metastatic lesion. To achieve this, the metastasis weighting parameters were automatically adjusted in the optimization framework. In the second set of optimization simulations, the weighting parameters were varied to prevent any necrotic damage to the spinal cord while maximizing the impact on the metastatic lesion.

**Figure 2:**
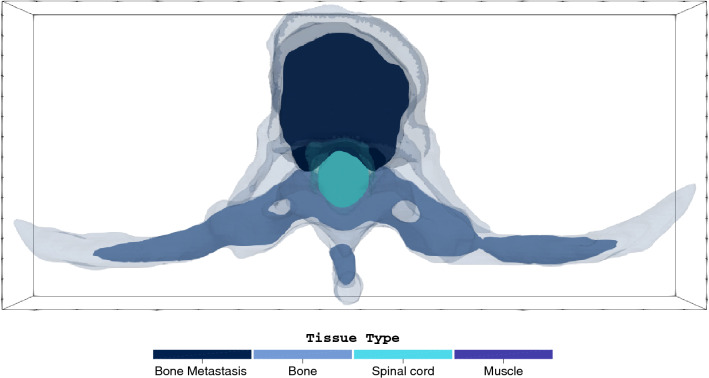
Central axial slice of the metastatically involved T8 vertebra with the surrounding healthy tissues. For treatment planning, the metastasis (dark blue) is considered the target and the spinal cord (light blue) is the primary organ at risk. For illustrative purposes, the bone is shown in light blue, and the muscle is made transparent.

**Figure 3:**
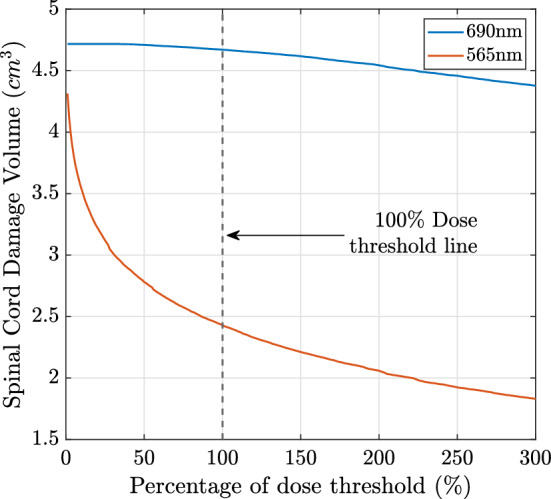
Dose-volume histogram of the spinal cord, for the 4 cut-end fibres configuration at the 690 nm and 565 nm wavelengths for treatment optimized to 90% tumour reduction.

### Simulations for cut-end fibre light delivery

**Figure 4:**
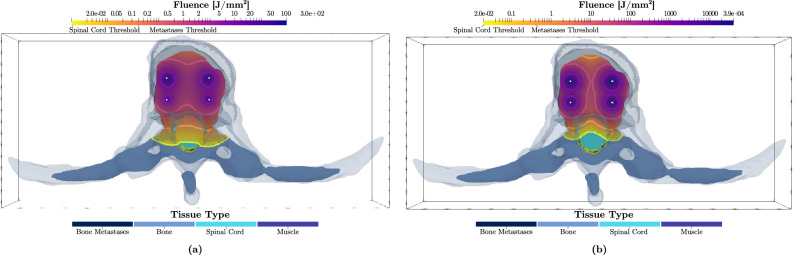
Optimized treatment plans for wavelengths of (**a**) 690 nm and (**b**) 565 nm using a 4-source configuration (4 cut-end fibres with $NA =0.22$ and $200~\upmu$m radius positioned at the center of the metastatic lesion). The resulting iso-fluence contours are overlaid on the 3D model, demonstrating the achievement of the minimum necrotic threshold within the metastatic region (0.4 J mm$^{-2}$) as per the colour map shown. The total energy at the 690 nm wavelength ($E_{total}$ = 1247.6 J) was distributed across the 4 cut-end fibres [ordered horizontally from top left to bottom right) as follows: $E_1=439.6$ J, $E_2=487.6$ J, $E_3=189.6$ J, $E_4=130.8$ J. At 565 nm, the total energy $E_{total}$ = 167.3 $\times 10^3$ J, distributed across the 4 cut-end fibres as: $E_1={64.6}\times 10^3$ J, $E_2={49.5}\times 10^3$ J, $E_3={32.4}\times 10^3$ J, $E_4={20.8}\times 10^3$ J.

Figure 4 shows the iso-fluence contours at the centre slice of the metastatic lesion (see Fig. 2) for both the 690 nm and 565 nm treatments. The yellow contour shows the necrotic threshold dose of the spinal cord (0.02 J mm$^{-2}$), while the orange contour indicates the necrotic threshold for the metastasis (0.4 J mm$^{-2}$). For the 690 nm treatment, the overall energy needed is estimated to be 1247.6 J. However, the potential overall damage to the spinal cord was around 4.7 cm$^3$, which is almost the entire segmented spinal cord tissue. The 565 nm treatment shows significantly reduced damage to the spinal cord (2.4 cm$^3$, representing ${\sim }\,50\%$ damage reduction). However, the estimated required energy for the 565 nm treatment is infeasible at 167.3 kJ. Figure 3 compares the spinal cord dose-volume histograms of the two treatments. While the 565 nm wavelength can significantly reduce the damage to the spinal cord, it would require a much longer treatment duration.

### Simulations for cylindrical diffusers light delivery

**Figure 5:**
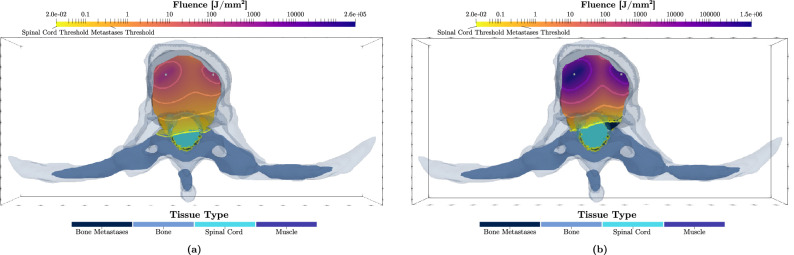
Optimized treatment plans for wavelengths of (**a**) 690 nm and (**b**) 565 nm using a 2-source configuration (2 cylindrical diffusers with a radius of 500 $\upmu$m). The resulting iso-fluence contours are overlaid on the 3D model, highlighting the the minimum necrotic threshold within the metastatic region (0.4 J mm$^{-2}$) as per the colour map shown. The total energy at the 690 nm wavelength ($E_{total} = 2914$ J) was distributed across the 2 light-emitting cylinders as: $E_1=1981.8$ J and $E_2=932.4$ J, for the left and right sources respectively. At 565 nm, the total energy was $E_{total} = {9.3}\times 10^6$ J, distributed as: $E_1={8.35}\times 10^6$ J and $E_2={0.95}\times 10^6$ J.

Similar to the cut-end fibres configurations, Fig. 5 shows the iso-fluence contours for a 2-cylindrical diffusers scenario at the two wavelengths. A similar trend is seen in that the 565 nm treatment significantly decreases the damage to the spinal cord. The damage reduction is more substantial than in the cut-end fibres scenario; the 565 nm wavelength yields a damage volume of only 1.4 cm$^3$ (70% damage reduction). However, the required energy (9.3 $\times 10^3$ kJ) would necessitate a much longer treatment duration.

### Summary of simulations

The above simulations were executed for the three metastatically involved vertebrae shown in Table 1. The results of the simulations for the metastatic lesion (volume and damage fraction (%)), the spinal cord (volume at risk and the damaged volume) and the required energy at 690 nm and 565 nm for a treatment optimized for 90% tumour destruction are shown in Table 4. Notice that for the small metastatic lesion in case 3, the 565 nm is effective for both cut-end fibres and cylindrical diffusers. For the cut-end fibres scenario, less than 18% of the spinal cord is damaged at 90% damage to the tumour with only 3.7 kJ needed.

**Table 4:** PDT induced tumour and spinal cord damage as a function of wavelength and photon source.

Case	Metastasis		Spinal cord				
	Volume (cm$^3$)	Damage (%)	Volume (cm$^3$)	Cut-end fibres		Cylindrical diffusers	
				Damage (cm$^3$)	Energy (kJ)	Damage (cm$^3$)	Energy (kJ)
1 @690 nm	23.21	90	4.72	4.7	1.25	4.6	2.91
1 @565 nm				2.4	167.3	1.4	$9.3\times 10^{3}$
2 @690 nm	8.84	90	10.88	9.1	0.43	9.9	0.26
2 @565 nm				2.8	49.97	3	27.65
3 @690 nm	4.35	90	8.79	6.7	0.15	6.7	0.15
3 @565 nm				1.51	3.68	1.18	6.79

To simulate a palliative treatment goal, the optimization was run focusing on spinal cord preservation. In this, the source placement remained fixed but the tissues’ weighting parameters in PDT-SPACE were adjusted to attain near-zero damage on the spinal cord. Table 5 reports the limited (0.04% to 15%) attainable bone metastasis destruction fraction under this scenario.

**Table 5:** PDT induced tumour volume reduction in the metastatically involved vertebrae as a function of wavelength and photon source for near complete preservation of the spinal cord.

Case	Spinal cord			Metastasis				
	Volume (cm$^3$)	Cut-end fibres	Cylindrical diffusers	Volume (cm$^3$)	Cut-end fibres		Cylindrical diffusers	
		Damage (cm$^3$)	Damage (cm$^3$)		Damage (%)	Energy (kJ)	Damage (%)	Energy (kJ)
1 @690 nm	4.72	0.01	0	23.21	2.1	0.008	1.5	0.005
1 @565 nm		0.04	0		15	0.36	13	0.416
2 @690 nm	10.88	0.002	0.27	8.48	0.06	0.002	0.04	0.001
2 @565 nm		0.02	0.003		2.4	0.038	1.7	0.013
3 @690 nm	8.79	0.0003	0.15	4.35	0.16	0.002	0.07	0.001
3 @565 nm		0.09	0.02		5.1	0.039	6.6	0.024

## Discussion

PDT is increasingly being utilized as a focal oncology therapy, with recent approvals for prostate and brain tumours in various jurisdictions including Europe, the USA and Japan. This is due to the non-toxicity of the photosensitizer drugs in the absence of light. Additionally, the short penetration of light in biological tissues makes PDT less invasive to the surrounding critical structures, making repeatability of the treatment in case of recurrent pain possible. In considering PDT for the treatment of spinal metastasis, the advantages of a localized non-thermal therapeutic approach must be weighed against any potential risk to the spinal cord. Predictive algorithms that minimize and quantify the risk of spinal cord damage based on each patient’s anatomy and tumour volume will be essential to achieve a broad acceptance for PDT in these critical situations and guide appropriate patient selection.

Modelling of light propagation in complex geometries with heterogeneous optical property distributions requires high-resolution clinical imaging and precise delineation of these structures. In this study, the tissue regions of interest were manually delineated (as is the convention in the clinical workflow of radiation therapy); however, several automated and semi-automated approaches for the segmentation of spinal images have been proposed and can be implemented to streamline the workflow for clinicians^40^.

The simulations provided here are based on assumed photodynamic threshold data from prior publications^38^ considering a least favourable responsivity difference between the spinal cord and the target metastasis, given the current lack of tissue specific PDT responsivity threshold values from the literature. The spinal cord mainly consists of a peripheral region that contains neuronal white-matter tracts. Internal to this region, there are neuronal grey-matter-like structures. White matter is rather resistant to PDT, while grey-matter is less resistant^38^. To be conservative, we assume in this work that the PDT threshold dose for the spinal cord is similar to that of grey-matter. In general, the threshold values of normal tissues are lower than those of malignancies, due to the latter’s ability to neutralize higher reactive oxygen species (ROS) concentration^41^. As such, the potential damage to the spinal cord reported in this study is likely an overestimate when optimizing to a level of 90% destruction of the metastatic lesion. Similarly, the maximum attainable malignancy destruction is likely underestimated in this model when optimizing based on complete spinal cord preservation. Experimental determination of PDT threshold values is underway in ongoing preclinical rat models^42^, in which the extent of the treatment effect is assessed through histology^38^. Preliminary results on $n=3$ T10 vertebrae cases show that the photodynamic threshold ranges between  0.61–2.36 $\times 10^{18}$ photons/cm$^3$, which is higher than the threshold assumed in this study. Using established tissue optical properties^31^, the PDT dose gradients per source can be calculated independent of the uncertainty in the PDT threshold values. However, the absolute power requirements, the total delivered energy and the energy distribution between the multiple sources would vary.

The following conclusions are not affected by the unknown PDT threshold values. Exploiting the strong light attenuation of the 565 nm excitation light aids in limiting damage to the spinal cord; however this required 134 and 3196 times more total energy to achieve the same fraction of tumour destruction for the cut-end and the cylindrical emitters, respectively (Figs. 4, 5). The higher optical energy delivery is partially due to the 5-times lower molar extinction coefficient at 565 nm versus 690 nm. Assuming the same power delivery, the added energy translates directly into a proportional increase in exposure time. Using cylindrical fibres instead of the cut-end fibres resulted in a 130% increase in total energy requirements (2.91 kJ versus 1.25 kJ). However, the power per optical fibre can be higher when using cylindrical diffusers versus cut-end fibres. Assuming a power delivery of 200 mW cm$^{-1}$ across the diffuser length, treatment time of ${\sim }\,41$ min would be required. It is worth mentioning, however, that depending on the blood volume in the tissues, the absorption coefficient, $\mu _a$, can vary significantly^35^. Our simulation results showed that for high blood content in the tumour, a solution may not be found for fixed, empirically determined fiber source positions as done in this work. This motivates the need for online dosimetry to recover the optical properties in real time similar to the work proposed by Swartling et al.^43^.

In any case, the PDT-SPACE automated plan can be tailored to clinical plans focused on high levels of tumour destruction (Table 4) or accommodate less comprehensive, palliative treatment, which prioritizes preservation of the spinal cord volume at risk (Table 5). For example, Table 5 shows that treatment at 565 nm for case number 1 with around the same total energy (0.36 kJ) given to the 690 nm treatment—that kills 90% of the tumour (case 1 in Table 4)—reduces the metastatic lesion volume by at least 15% with no damage to the spinal cord.

Ultimately, treatment planning using the PDT-SPACE platform may allow a combination of different treatment wavelengths to be assessed. Future investigations may consider a 565 nm source positioned in the posterior aspect of the vertebral body (closer to the spinal cord), and a 690 nm source in a more anterior location. This would reduce the overall optical energy requirements and treatment time, enabling a safe, feasible and effective approach to PDT tumour ablation in the spine, specifically when there is a risk of cortical breach of the vertebral body towards the spinal cord. Another possible future direction is the use of a combination of BPD-MA and lipid-anchored BPD to achieve photo-damage at a lower light dose by targeting mitochondria, ER and lysosomes simultaneously as shown by Rizvi et. al.^44^ in an in-vitro ovarian cancer 3D model. In principle, this would allow achieving metastatic damage at lower energy levels even for the shorter wavelength treatment. However, more pre-clinical and clinical studies are needed to determine the feasibility of this approach and to evaluate the lower PDT dose thresholds required by PDT-SPACE.

## Data Availability

The datasets generated and/or analysed during the current study available from the corresponding author on reasonable request.
